# Comparative Analysis of Actual Cost and Indonesian Case-Based Groups Rate in Inpatient Suspect of Acute Hepatitis of Unknown Aetiology

**DOI:** 10.21315/mjms-05-2025-321

**Published:** 2026-02-28

**Authors:** Siti Maemun, Aninda Dinar Widiantari, Nur Aliza, Vivi Setiawaty, Lhuri Dwianti Rahmartani

**Affiliations:** 1 Sulianti Saroso Infectious Disease Hospital, Tanjung Priok, North Jakarta, Indonesia; 2 Faculty of Health Science, University of Respati Indonesia, Cipayung, East Jakarta, Indonesia; 3 Indonesian College of Epidemiology, Kebayoran Baru, South Jakarta, Indonesia; 4 Faculty of Public Health, Universitas Indonesia, Depok, West Java, Indonesia

**Keywords:** cost control, diagnosis, INA-CBG, suspected AHUA

## Abstract

**Background:**

Hospitals are crucial in implementing the referral and National Health Insurance (NHI) systems. The reimbursement structure by the Social Security Administrator for Health (SSAH) relies on the Indonesian Case-Based Groups (INA-CBG) method. This study aims to discern the variance between hospital inpatient costs and the corresponding inpatient INA-CBG rates, specifically for patients with suspected acute hepatitis of unknown aetiology (AHUA).

**Methods:**

This research used a descriptive cross-sectional method. We compared the actual hospitalisation cost and INA-CBG’s tariff in an Indonesian tertiary hospital from July to December 2022. The study encompasses patients with AHUA and uses a saturation sampling method.

**Results:**

Of the 17 patients suspected of AHUA, 16 made payments through NHI. A comprehensive analysis reveals that the total hospital costs based on INA-CBG rates show a deficit of IDR 21,604,464, reflecting a difference of 13.96%. The primary diagnoses among patients vary, with presentations of a combination of viral, bacterial, and parasitic infections alongside bile or liver disorders. The hospital incurred losses from services rendered to 10 patients. Notably, the significant allocations of hospital costs include laboratory (46.84%), pharmacy (19.01%), and accommodation (17.28%).

**Conclusion:**

To maintain effective cost control in inpatient services, the focus should prioritise medical appropriateness, explicitly referencing Clinical Practice Guidelines (CPG) and Clinical Pathways.

## Introduction

Emerging infectious diseases (EIDs) have garnered focused attention in Indonesia, as they pose a significant public health concern. Approximately 70% of EIDs affecting humans have a zoonotic origin (1). The Ministry of Health documented SARS cases in 2005, H1N1, SARS, H5N1, Zika virus, COVID-19, monkeypox, and acute hepatitis of unknown aetiology (AHUA) in Indonesia. As of 8 July 2022, the World Health Organization received reports of 1,010 probable AHUA cases from 35 countries (2). By 12 March 2023, Indonesia had reported 117 cases (48 probable, 8 pending, and 61 discarded cases) across 22 provinces. Among these, the Jakarta Greater Area emerged as a significant contributor to probable AHUA cases, accounting for 15 cases (3). Sulianti Saroso Infectious Disease Hospital (SSIDH) reported one probable and 16 discarded cases.

Being designated as the coordinator of the EID management network (4), SSIDH serves as a tertiary hospital with elevated morbidity, mortality, and financing rates. Successful implementation necessitates high competence in human resources, infrastructure, equipment, and other resources, all aligned with established standards. Furthermore, SSIDH has been appointed as a centre of excellence for infectious diseases (5), tasked with orchestrating comprehensive individual health services, specifically in the infectious disease domain. These dual mandates guide SSIDH in delivering services to the community, particularly in infectious disease management.

Indonesia launched the National Health Insurance (NHI) programme in 2014, intending to cover 98% of its population by 2024 (6). The NHI scheme in Indonesia operates by collecting contributions from formal, informal, and non-salaried workers. It also pays full or partial premiums for low-income members, ensuring broad coverage. As of 2019, the NHI covered approximately 84% of Indonesia’s population (7). In addition to the Social Security Administrator for Health (SSAH), Indonesia’s health system includes other government schemes financed through taxes. Non-public schemes include company health coverage, private health insurance, and out-of-pocket (OOP) payments. In 2019, the government’s spending on health in Indonesia reached IDR 113.6 trillion (approximately USD 7.9 billion), nearly doubling the amount spent in 2015 (8, 9). While reliance on OOP payments has decreased in recent years, it is notable that around one-third of health expenditure in Indonesia still comes from OOP payments. According to Indonesia’s National Health Accounts data 2019, OOP payments were the most significant contributor to total health expenditure, accounting for 32.1%. Government schemes and the NHI represent substantial portions of total health expenditure, accounting for 29.1% and 23.1%, respectively. Health expenditure through private health insurance is relatively low, comprising only 3.5% of the total health expenditure.

As mandated by the 58th World Health Assembly in 2005, the WHO encouraged every country to develop universal health coverage to ensure access to essential health services for its entire population (10, 11). In alignment with the Regulation of the Minister of Health Number 69 of 2013, which outlines health service tariff standards at first level health facilities and advanced health facilities, SSAH utilises the INA-CBG’s method to remunerate hospitals for health services provided to participants. The INA-CBG’s tariff represents the claim payment amount disbursed by SSAH to advanced health facilities for service packages based on diagnosis classification. The significance of this disease classification is underscored, by the fact that variations in health financing exist even when individuals share the same diagnosis.

Nevertheless, the effectiveness of the current INA-CBG system has been questioned. Research findings indicate a tendency for INA-CBG rates to surpass service fees, particularly in nonsurgical cases. Conversely, for surgical cases, the trend in INA-CBG rates is observed to be lower than the service fee. Moreover, according to Puspandari et al. (12), health financing is influenced by various factors, such as drug costs (13), duration of hospitalisation (14), comorbidities (15), complications (15), utilisation of the intensive care unit (ICU), and hospital location (12). Additionally, research conducted by Ambarriani (16) indicates that inpatient class (12, 17, 18) and disease severity are also correlated with health financing. Notably, the cost attributed to catastrophic diseases accounts for 32% of the total health expenditure.

Hospitals that manage to decrease production costs for specific diagnostic group medical services, resulting in an average unit cost per patient lower than the INA-CBGs rate, can benefit from the health services provided. Conversely, the hospital will incur a financial loss if the unit cost per patient exceeds the INA-CBGs rate. Given this background, it becomes crucial to investigate whether there is a disparity in the average actual hospitalisation cost at the hospital with INA-CBG’s inpatient rate, as determined by SSAH. This inquiry is particularly relevant in light of the roles and responsibilities of SSIDH in managing EIDs and this context-specific AHUA.

## Methods

### Data Sources

The study was conducted from July to December 2023 at SSIDH in Jakarta, an Indonesian tertiary hospital. The data sources encompass electronic medical records containing patient characteristics, symptoms and signs, laboratory parameters, INA-CBG, and hospital tariff information (in Indonesian Rupiah). According to Ministry of Health Regulation No. 6 Year 2018, INA-CBG rates were paid based on ICD, following a paediatrician’s final primary and secondary diagnosis.

### Analytical Approach

This research adopts a quantitative approach, employing a descriptive research design with a cross-sectional method. The study focuses on the population of suspected AHUA (19) inpatient children (≤ 16 years old) (20, 21) with aspartate aminotransferase (AST) or alanine aminotransferase (ALT) level > 500 IU/L (19–22) who were admitted under the NHI programme. The study protocol was approved by the Health Research Ethics Committee of SSIDH under reference number 13/XXXVIII.10/II/2023.

## Results

### Descriptive Analysis

Out of the 17 suspected AHUA patients, 16 were covered by the NHI. Most of these patients were female, accounting for 62.5%, and the predominant age group was over five years, constituting 81.2%. Furthermore, a significant portion of the patients resided in Jakarta. For patients treated with symptoms and clinical signs of acute hepatitis, a considerable proportion exhibited symptoms such as fever (75.0%) and nausea/vomiting (81.2%). Icteric clinical signs were also observed in 43.8% of the cases. Given SSIDH’s role as an infectious disease referral hospital, most patients (62.4%) were referred from hospital type C or D, while 18.8% were referred from primary health care. Out of the 16 patients, one (6.3%) died due to the illness (Table 1).

The primary clinical criteria for suspected AHUA patients were evaluated based on AST and ALT parameters exceeding 500. The average AST was 598.31 (SD = 563.30), and the average ALT was 604.69 (SD = 361.92). Additionally, the average length of stay (LoS) for these patients was five days (SD = 2.70) (Table 1).

The total hospital cost differed significantly from the INA-CBG tariff, with a negative discrepancy of IDR 21,604,464 (−13.9%), indicating that the actual hospital cost exceeded the INA-CBG reimbursement rate. The INA-CBG diagnosis groupings varied. Patients classified under viral, bacterial, or parasitic infections with severe or moderate INA-CBG severity levels, combined with bile or liver disorders classified as mild severity, exhibited a negative cost difference. The hospital incurred losses from the services provided to 10 patients, as outlined in Table 2.

The laboratory received the most substantial average allocation of hospital costs (46.1%), followed by accommodation (18.2%) and pharmacy (17.3%) (Table 3).

## Discussion

Financial administration data from SSIDH reveal that the hospital incurred losses in 2022 when it hospitalised 16 suspected AHUA cases. The variance between hospital rates and INA-CBG rates paid by SSAH raises concerns for hospital management. This potential loss risks the quality of service provided, particularly for inpatients requiring intensive monitoring and prompt, accurate treatment. Addressing this discrepancy becomes imperative to ensure the sustained delivery of high-quality healthcare services (23).

Following the Health Ministerial Regulation of the Republic of Indonesia, Number 59 of 2016, EIDs pose a significant threat to global health security, as they can lead to outbreaks and potentially cause public health emergencies of international concern (PHEIC) (24, 25). These events not only result in loss of life (26) but also entail substantial economic losses (27). AHUA is recognised as a new EID, and the state assumes full responsibility for the comprehensive management of EID patients. Specific fee exemptions for EIDs encompass service administration costs, services and care in the emergency room, isolation/ICU room utilisation, doctor services, supporting diagnostic examinations, medications and medical devices, consumables medical supply, hospital referring fee, and corpse management. The fee waiver applies to patients treated at national/provincial/regional referral hospitals and other hospitals designated by the Minister.

According to Mercer Marsh Benefits’s 2022 Cost of Care Report, infectious and parasitic diseases constitute Indonesia’s most common disease groups. The predominant diagnoses include dengue fever (25%), gastroenteritis, and other colitis (21%). This specific group of infectious and parasitic diseases also incurs the highest costs. Notably, COVID-19 is the highest-cost diagnosis within this group, accounting for 40% of the associated costs (28).

In its capacity as coordinator of the EID syndrome surveillance network hospitals (4), a national referral hospital for infectious disease (29), and the National Infectious Disease Centre of Excellence, SSIDH plays a pivotal (30). The hospital must consistently provide patient services for EIDs and other infectious and noninfectious diseases (31). Consequently, hospital preparedness is essential to facilitate fast, precise, high-quality EID response, thereby reducing the threat of patient mortality (32, 33). Fundamental requirements include reliable human resources, comprehensive supporting examinations, sample logistics, and infrastructure that meets the criteria for effective case management, encompassing the EID response in hospitals (32, 34). These factors ensure that the hospital can respond effectively to EIDs and contribute to public health safety. The most significant proportion of the cost of hospital care for patients with new infectious diseases are laboratory, accommodation, and pharmacy (drug fee and consumable medical supplies) (35, 36). The study only explored the health cost of inpatient cases of suspected AHUA in SSIDH.

The INA-CBG is a reimbursement system based on grouping disease diagnoses. This payment mechanism is derived from a case-mix system that groups diagnoses/symptoms and procedures sharing similar clinical characteristics and costs, utilising groupers for classification. INA-CBG payments are implemented across hospitals A, B, C, and D, as well as general and national referral hospitals. Hospitals receive payments based on the INA-CBG tariff, representing the cost incurred for the specific diagnosis. The INA-CBG rates within the National Health Coverage programme are formulated using cost data from 137 government and private hospitals and coding data for 6 million diseases (36). Several factors influence the amount of INA-CBG costs, including the primary diagnosis, secondary diagnoses, comorbidities or complications, severity, specific medical procedures and interventions, and the patient’s age (37–39). Payments under INA-CBG are made per healthcare episode, covering a series of patient care activities until completion. This payment package encompasses doctor consultations, supporting examinations, medications, consumable medical supplies, accommodation, and other costs related to patient health services. The INA-CBG pattern provides a comprehensive approach to payment, covering various aspects of patient care within a specific episode.

A notable discrepancy exists between hospital and SSAH rates, primarily stemming from the different payment methods. Hospitals typically use Fee-for-Service, a retrospective payment method that determines fees after services are provided. In contrast, SSAH adopts the INA-CBG tariff system, a prospective payment method in which health service rates are established before patient services are rendered. As identified in hospital research, various factors contribute to the differences in rates. These factors include the standard real rate of the hospital, the type and location of the hospital, the precision of ICD-10 coding, LoS, disease severity, class of care, and the number of diagnosed complications.

Clinical chemistry examinations reveal a pattern where, as patients’ conditions worsen, the proportion of examination costs decreases. This trend is also observed in routine radiological, urine, and stool examinations. In contrast, haematology, microbiology, and immunology/serology examinations demonstrate a different pattern, wherein the more severe the patient’s condition, the higher the proportion of examination costs.

It is imperative to enhance efficiency and control costs by developing a comprehensive system to mitigate these disparities. This involves the compilation of Clinical Practice Guidelines (CPG), Clinical Pathways, and the Hospital Formulary. After the creation of these three documents, the establishment of a control team becomes essential. This control team should foster collaboration among all stakeholders in the field and ensure adherence to the established guidelines. Subsequently, the control team should conduct utilisation studies and analyses every month, displaying the cases handled for review.

The analysis revealed that the INA-CBG rate was lower than the cost of medical services for AHUA suspect cases, leading to financial losses for the hospital. Conversely, if the INA-CBG rate surpasses the cost of medical services at the hospital, it results in economic gains. Therefore, hospitals need to exercise caution in accurately calculating the cost of medical services to ensure their financial viability by generating surpluses or profits, while still maintaining the social function of the hospital (40, 41) and prioritising the referral of infectious diseases for national security (41). The revenue derived from health services to patients is the primary source of hospital financing and is essential in effectively managing hospital operations (23).

### Limitations

The researchers utilised secondary data and did not conduct a review of the case-mix process (healthcare service financing) on the subjects. The findings of this research are not representative of similar research in other hospitals, given that the case-mix process relies on the CPG, Clinical Pathways, and the Hospital Formulary applicable in each hospital.

## Conclusion

Financial losses from suspected AHUA cases reached 14% in 2022, necessitating careful attention. Addressing and reducing this rate must be a top priority for completion. Cost control in inpatient services should continue to prioritise medical fairness, guided by the CPG and Clinical Pathway. This presents a challenge for SSIDH to promptly establish CPGs and Clinical Pathways tailored for EIDs that are currently developing.

## Figures and Tables

**Table 1:** Characteristics of suspect AHUA

Characteristics	*n* (%)
**Sociodemographic**	

Sex	
Male	6 (37.5)
Female	10 (62.5)

Age	
0 to 5 years	3 (18.8)
> 5 years	13 (81.2)

Domicile	
South Jakarta	1 (6.3)
North Jakarta	6 (37.5)
West Jakarta	1 (6.3)
East Jakarta	2 (12.6)
Central Jakarta	1 (6.3)
Others	4 (25.0)

**Symptoms**	

Fever	
Yes	12 (75.0)
No	4 (25.0)

Nausea/vomiting	
Yes	13 (81.2)
No	3 (18.8)

Diarrhoea	
Yes	5 (31.3)
No	11 (68.7)

Stomachache	
Yes	8 (50.0)
No	8 (50.0)

Icteric	
Yes	7 (43.8)
No	9 (56.2)

Dark urine	
Yes	3 (18.8)
No	13 (81.2)

Referral	
Primary health care	3 (18.8)
Hospital	10 (62.4)
None	3 (18.8)

Outcome	
Death	1 (6.3)
Fully recovered	14 (87.6)
Returning home at the patient’s request	1 (6.3)
LoS^a^	5 (2.70)
AST^a^	598.31 (563.30)
ALT^a^	604.69 (362.92)

LoS = length of stay; AST = aspartate aminotransferase; ALT = alanine aminotransferase;

a mean (SD); SD = standard deviation

**Table 2:** INA-CBG, cost, and diagnosis

Subject	INA-CBG	INA-CBG budget (IDR)	Real cost (IDR)	Primary diagnosis*	Secondary diagnosis 1*	Secondary diagnosis 2*	Secondary diagnosis 3*	Secondary diagnosis 4*	Discrepancy (IDR)
1	Viral and other nonbacterial infections (severe)	2,483,500	4,285,849	A91	R74.0	-	-	-	−1,802,349
2	Viral and other nonbacterial infections (severe)	2,069,600	2,933,722	A91	R94.5	-	-	-	−864,122
3	Bile duct obstruction (mild)	8,157,300	18,275,589	K83.1	R74.0	-	-	-	−10,118,289
4	Certain infectious and parasitic diseases (moderate)	3,901,100	22,151,619	A01.0	K76.9	E87.6	E87.1	-	−18,250,519
5	Abdominal pain and gastroenteritis (moderate)	2,950,600	1,790,880	A09.9	A90	-	-	-	1,159,720
6	Certain infectious and parasitic diseases (moderate)	5,461,500	6,223,807	A49.9	K76.9	-	-	-	−762,307
7	Other hepatic disease (moderate)	6,795,000	3,424,066	B15.9	B86	-	-	-	3,370,934
8	Viral and other nonbacterial infection (mild)	2,069,600	5,393,270	A91	R74.0	-	-	-	−3,323,670
9	Gastrointestinal infections (moderate)	6,834,400	12,361,217	K65.9	K76.9	-	-	-	−5,526,817
10	Other hepatic disease (moderate)	8,166,000	10,304,728	B19.9	-	-	-	-	−2,138,728
11	Abdominal pain and gastroenteritis (moderate)	3,442,400	19,817,371	A08.0	E86	K71.6	-	-	−16,374,971
12	Other hepatic disease (moderate)	6,795,000	3,084,009	B15.9	K83.1	-	-	-	3,710,991
13	Other hepatic disease (moderate)	6,795,000	3,545,979	B15.9	K83.1	-	-	-	3,249,021
14	Viral and other nonbacterial infections (severe)	6,144,700	7,215,749	A91	K76.9	R57.1	-	-	−1,071,049
15	Other hepatic disease (mild)	5,832,900	4,468,829	B15.9	-	-	-	-	1,364,071
16	Non-complex respiratory system procedure (severe)	55,237,300	29,463,680	B19.0	J15.8	A41.9	R57.2	J80	25,773,620
**Total**		133,135,900	154,740,364						−21,604,464

* In International Classification of Diseases (ICD);

A01.0 = typhoid fever; A08.0 = rotaviral enteritis; A09.9 = gastroenteritis and colitis of unspecified origin; A41.9 = billable diagnosis code for sepsis, unspecified organism; A49.9 = bacterial infection, unspecified; A90 = dengue fever; A91 = dengue haemorrhagic fever; B15.9 = hepatitis A without hepatic coma; B19.0 = unspecified viral hepatitis with hepatic coma; B19.9 = unspecified viral hepatitis without hepatic coma; B86 = scabies; E86 = dehydration; E87.1 = hypo-osmolality and hyponatraemia; E87.6 = hypokalaemia; J15.8 = pneumonia due to other specified bacteria; J80 = acute respiratory distress syndrome; K65.9 = peritonitis, unspecified; K71.6 = toxic liver disease with hepatitis; K76.9 = liver disease, unspecified; K83.1 = obstruction of bile duct; R57.1 = hypovolemic shock; R57.2 = septic shock; R74.0 = nonspecific elevation of levels of transaminase and lactic acid dehydrogenase [LDH]; R94.5 = abnormal results of liver function; Secondary diagnoses 1, 2, 3, and 4 refer to other conditions diagnosed by the attending physician based on clinical symptoms. These secondary diagnoses may represent either complications or comorbidities.

**Table 3:** List real cost

Subject	Real cost (IDR)	LoS (days)	Accommodation (%)	Nursing fee (%)	Medication and CMS (%)	Lab (%)	Radiology (%)	Doctor fee (%)	Non-surgical procedure (%)	ECG (%)
1	4,285,849	3	10	1	17	61	0	5	5	0
2	2,933,722	5	21	6	15	36	0	9	12	0
3	18,275,589	8	17	2	4	69	2	6	0	0
4	22,151,619	9	7	4	13	72	1	3	0	0
5	1,790,880	1	24	9	4	42	0	20	0	0
6	6,223,807	5	31	8	8	43	0	9	0	0
7	3,424,066	5	14	9	17	43	4	13	0	0
8	5,393,270	5	5	5	26	59	0	4	1	0
9	2,361,217	8	26	9	23	31	5	6	0	0
10	0,304,728	12	26	9	27	24	4	9	0	1
11	9,817,371	5	10	4	7	73	0	6	0	0
12	3,084,009	4	21	5	14	47	0	11	0	2
13	3,545,979	4	18	5	13	51	0	12	0	0
14	7,215,749	3	17	6	37	32	0	8	0	0
15	4,468,829	4	21	5	15	40	7	11	0	1
16	29,463,680	4	23	5	37	13	1	4	17	0

Total	154,740,364	5*	18.2*	5.8*	17.3*	46.1*	1.5*	8.7*	2.2*	0.3*

LoS = length of stay; CMS = consumable medical supplies; ECG = electrocardiography;

* Mean
